# Potassium Deficiency Significantly Affected Plant Growth and Development as Well as microRNA-Mediated Mechanism in Wheat (*Triticum aestivum* L.)

**DOI:** 10.3389/fpls.2020.01219

**Published:** 2020-08-14

**Authors:** Thomas Elliott Thornburg, Jia Liu, Qian Li, Huiyun Xue, Guo Wang, Lijie Li, Julia Elise Fontana, Kyle E. Davis, Wanying Liu, Baohong Zhang, Zhiyong Zhang, Mingjiu Liu, Xiaoping Pan

**Affiliations:** ^1^Henan Collaborative Innovation Center of Modern Biological Breeding and Henan Key Laboratory for Molecular Ecology and Germplasm Innovation of Cotton and Wheat, Henan Institute of Science and Technology, Xinxiang, China; ^2^Department of Biology, East Carolina University, Greenville, NC, United States; ^3^College of Life Sciences, Anhui Normal University, Wuhu, China

**Keywords:** potassium deficiency, abiotic stress, wheat, microRNA, chlorophyll

## Abstract

It is well studied that potassium (K^+^) deficiency induced aberrant growth and development of plant and altered the expression of protein-coding genes. However, there are not too many systematic investigations on root development affected by K^+^ deficiency, and there is no report on miRNA expression during K^+^ deficiency in wheat. In this study, we found that K^+^ deficiency significantly affected wheat seedling growth and development, evidenced by reduced plant biomass and small plant size. In wheat cultivar AK-58, up-ground shoots were more sensitive to K^+^ deficiency than roots. K^+^ deficiency did not significantly affect root vitality but affected root development, including root branching, root area, and root size. K^+^ deficiency delayed seminal root emergence but enhanced seminal root elongation, total root length, and correspondingly total root surface area. K^+^ deficiency also affected root and leaf respiration at the early exposure stage, but these effects were not observed at the later stage. One potential mechanism causing K^+^ deficiency impacts is microRNAs (miRNAs), one important class of small regulatory RNAs. K^+^ deficiency induced the aberrant expression of miRNAs and their targets, which further affected plant growth, development, and response to abiotic stresses, including K^+^ deficiency. Thereby, this positive root adaption to K^+^ deficiency is likely associated with the miRNA-involved regulation of root development.

## Introduction

Wheat (*Triticum aestivum* L.) is one of the most important crops in the world, which counts about 35% of the staple food for the world’s population (Paux et al., 2008). The problem of potassium (K^+^) deficiency in agriculture has a remarkably wide reach, affecting crops around the world, including wheat (Ruan et al., 2015). Studies on K^+^ concentration in the soil have revealed that as much as 75% of paddy soils are K^+^ deficient in China and that in Southern Australia is 67% (Rengel and Damon, 2008; Ruan et al., 2015). Generally, soil K^+^ levels are found below 0.3 mM in these fields (Ruan et al., 2015). Given the prevalence of agricultural plots across the world with a K^+^ deficiency problem, more research should be conducted for developing strategies to address this issue.

K^+^ is a crucial nutrient in many plant processes including photosynthesis, osmoregulation, and enzyme activations in many metabolic pathways (Maathuis, 2009). K^+^ regulates enzymes both transcriptionally and post-transcriptionally (Maathuis, 2009). It plays important roles in plant growth and development making it a point of interest to maximize crop yield (Pettigrew, 2008). Research has shown that the deficiency of K^+^ in plants is a form of abiotic stress that triggers a variety of responses resulting in reduced growth and productivity (Hafsi et al., 2014). Plant coping strategies such as morphological and physiological modifications and regulation of K^+^ transport systems exist; however, molecular mechanisms of adaption to K^+^ deficiency have not been fully understood (Hafsi et al., 2014). By further investigating the effects of K^+^ deficiency on plants and identifying the mechanisms involved in driving the observed changes, crop yields and quality can be maximized.

Root morphology change is one of the vital plant responses to K^+^ efficiency, manifesting commonly as root growth retardation (Hermans et al., 2006; Jordan-Meille et al., 2018). However, the impact varies among different plant species, genotypes, the degree and duration of the deficiency, and culturing conditions. In rice, a resistant genotype presented as a higher abundance of ﬁne roots within the root system under K^+^ deprivation (Jia et al., 2008; Jordan-Meille et al., 2018). In *Arabidopsis thaliana*, the number and length of the ﬁrst order lateral roots were decreased, while the number of the second order laterals was increased in K^+^ depleted plants (Kellermeier et al., 2014). Additionally, the elongation of the main roots was inhibited (Shin and Schachtman, 2004; Jung et al., 2009; Kim et al., 2010; Kellermeier et al., 2013). In cotton, K^+^ deficiency inhibited lateral root formation and elongation, and retarded main root growth after 4 DAT of the treatment (Zhang et al., 2009). Although it is well studied that K^+^ deficiency induced aberrant growth and development of plant and altered the expression of protein-coding genes, more systematic investigations on root development under K^+^ deficiency and the underlying molecular mechanisms are needed.

MicroRNAs (miRNAs) are small regulatory RNA molecules that bind to target mRNA sequences and form a mRNA-induced gene silencing complex (RISC) (Li and Zhang, 2016). They are a class of small non-coding RNAs responsible for regulating target genes in many metabolic and developmental processes (Li and Zhang, 2016; Jaubert-Possamai et al., 2019). miRNAs are approximately 20–22 nucleotides in length and are processed by *Dicer-like 1* (*DCL1*) enzyme from precursor sequences (pre-miRNAs) in plants. miRNAs are highly evolutionarily conserved from species to species (Zhang et al., 2006); many miRNAs are involved in the regulation of plant response to abiotic and biotic stresses (Zhang, 2015; Jaubert-Possamai et al., 2019). Gene expression is inhibited by miRNAs in two ways: either by the targeted degradation of mRNA molecules or by inhibition of protein translation. Research identifying new miRNAs and determining their functions is growing rapidly in a multitude of plant species (Zhang and Wang, 2015). There are many reports on the role of miRNAs in plant root growth and development (Khan et al., 2011; Couzigou and Combier, 2016; Zhang and Unver, 2018). Several miRNAs were found to regulate root structure change induced by phosphorus or nitrogen deficiency (Nguyen et al., 2015). However, the study of miRNA expression during root development change under K^+^ deficiency condition is rare, if any. In this study, we not only studied the impact of K^+^ deficiency on wheat seedling growth and development but also systematically studied the effect of K^+^ deficiency on wheat root development and morphology. Our results show consistent findings with other studies in that K^+^ deficiency affected wheat seedling growth and development. We also found that although K^+^ deficiency did not affect root vitality, it affected root development, including root branching, root area, and root size. Importantly, we found that K^+^ deficiency altered the expression of miRNAs and their targets; the majority of gene targets regulated by miRNAs are related to root development. Manipulating the expression of these miRNAs and their targets may become a new strategy to increase plant biomass and yield under potassium K^+^ condition and enhance the fertilizer efficiency.

## Materials and Methods

### Wheat Seed Sterilization and Growth Conditions

Wheat (*Triticum aestivum* L.) cultivar AK-58 was used to test the impact of K^+^ deficiency. AK-58 was bred by the Henan Institute of Science and Technology, which is the No. 1 widely-adopted wheat cultivar by farmers in China. AK-58 has many elite traits, including high yield, high quality, and tolerance to both abiotic and biotic stresses; however, it is sensitive to NPK deficiency and requires substantial fertilizers. Mature wheat seeds were collected and surface sterilized in 9% H_2_O_2_ solution for 5 min followed by 5 times of washes with sterilized water. Sterilized seeds were then carefully placed at ~1 cm apart with a 5-cm space from the top of the germination paper soaked in a nutrient solution with half in control solution and half in K^+^ deficiency solution. The papers were rolled and placed in a plastic tub of nutrient solution with half in control solution and half in K^+^ deficiency solution in the dark at 28°C for six days until seedling emergence was observed. Tubs were covered with a plastic sheet punctured with a needle to prevent evaporation of solution while allowing airflow.

After six days of germination, 25 seedlings, respectively, from control and K^+^ deficiency were collected and transferred to a hydroponic system of corresponding control or K^+^ deficiency under lamplight at 28°C. The transferring day was defined as 0 day after transferring (0 DAT). The system contained a nutrient solution that had 2 mM NaCl, 2.5 mM Ca(NO_3_)_2_, 0.5 mM NH_4_H_2_PO_4_, 1 mM MgSO_4_, 0.1 mM EDTA FeNa, 0.02 mM H_3_BO_3_, 0.001 mM MnSO_4_, 0.2 μM CuSO_4_, and 0.005 μM (NH_4_)_6_Mo_7_O_24_•4H_2_O. The K^+^ concentrations were manipulated to create a normal control of 2.5 mM KCl and a K^+^ deficiency treatment with low K^+^ concentration of 0.02 mM KCl and NaCl at 2.48 mM.

### Root Morphology Analysis

At 4 DAT and 8 DAT of K^+^ treatment, the plant morphology was observed, and the number of roots was accounted. After that, the whole plants and each part of the plants were scanned by using an EPSON Scanner, and pictures were taken. The software WinRHIZO was employed to analyze the root length, root area, root volume, and root dimeter. The length of the seminal roots and the length of roots that had not branched roots were also measured.

### Dry Biomass Measurement

After pictures were taken and morphological characteristics were recorded, both up-ground shoots and under-ground roots were placed in an oven with 80°C for drying. After 4 days of drying, the dry mass was measured and calculated.

### Root and Leaf Respiration Measurement

After 4 DAT and 8 DAT of K^+^ treatment, six wheat seedings were randomly selected from the K^+^ deficiency treatment and the controls, respectively. The middle 2 cm of the first fully opened leaf and root section (5–7 cm from the stem) was cut and weighted. Then, the samples were placed into the incubator chamber of the Clark Chlorolab2 system followed by adding 2 ml saturated CaSO_4_ solution. The Clark Chlorolab2 was employed to measure the consumption of O_2_ for 10 min. The consumption rate of O_2_ was calculated. Six biological replicates were measured for each tissue of each treatment at each time point.

### Root Vigor Measurement

Root vigor was measured by using the TTC method. Briefly, roots of each plant were weighted and cut into small fragments of ~1 cm in length followed by soaking in 0.6% TTC solution prepared by using 0.1 M phosphate buffer solution (PBS). After 24 h of dyeing by TTC, the root samples were washed three times with ddH_2_O. Finally, the stained roots were soaked in 95% ethanol for 10 min at an 85°C water bath to extract triphenylformamidine (TTF). Root vigor was measured using a spectrophotometer at 485 nm. The OD value was recorded. Six biological replicates were performed for each treatment at each time point.

### Measurement of Chlorophyll

After 4 DAT and 8 DAT of K^+^ treatment, the first fully-opened leaf was selected from each treatment and the control plant. The whole leaves were broken in a mortar with a small amount of CaCO_3_ and 2 ml acetone. Then, broken leaves were transferred into a 15 ml centrifuge tube. The leaf residue on the mortar was rinsed off using 3 ml acetone and then transferred into the centrifuge tube. The centrifuge tubes were centrifuged for 10 min at 10,000g. After adding acetone to the total of 5 ml, the chlorophyll content was measured using a spectrophotometer at wavelength of 663 and 645 nm. Six biological replicates were measured for each treatment at each time point.

### RNA Extraction, Reverse Transcription, and Gene Expression Analysis

Total RNAs were extracted using the MirVana miRNA Isolation Kit according to the manufacturer’s protocol. Briefly, after 4 and 8 DAT of treatment, root samples from the K^+^ deficiency treatment group and the control group were collected and immediately frozen in liquid nitrogen and then stored at −80°C until RNA extraction. About 200 mg of frozen root samples was used to extract total RNAs from each sample. Three biological replicates were performed for each treatment and the controls. Extracted RNAs were quantified, and the quality was tested using a Nanodrop ND-1000 (Nanodrop Technologies, Wilmington, DE). Extracted RNA was stored at −80°C until further analysis.

Reverse transcription of isolated RNA was performed following the manufacturer’s protocol of the TianGen miRcute miRNA First-strand cDNA Synthesis Kit (Beijing, China). miRNA specific stem-loop primers were used to synthesize cDNA from previously extracted RNA samples. Synthesized cDNA was then used in qRT-PCR.

Gene expression analysis of miRNAs and their targeted genes was performed by qRT-PCR following the manufacturer’s protocol as outlined in the TianGen miRcute Plus miRNA qPCR Detection Kit. The previously synthesized cDNA was amplified and quantified to determine the expression levels. The 50× ROX Control Dye and 50×ROX Reference Dye were used along with SYBR Green to facilitate the measurement of the expression levels. Expression levels were normalized against the reference gene *EF1A*. Fold change was calculated using the ΔΔC_T_ method. Three biological and three technical replicates were run for each gene and miRNA.

K^+^ deficiency affected root development in a wide range of plant species (Jia et al., 2008; Maathuis, 2009; Zhang et al., 2009; Hafsi et al., 2014; Guo et al., 2019). To study the potential miRNA-mediated mechanism in which plant responds to K^+^ deficiency, we have selected and tested a total of 20 miRNAs that are associated with root development (Khan et al., 2011; Couzigou and Combier, 2016; Li and Zhang, 2016) and responses to nutrients (Maathuis, 2009; Kulcheski et al., 2015) including nitrogen and phosphorus deficiency (Bao et al., 2019; Filina et al., 2019; Yang et al., 2019; Asefpour Vakilian, 2020; Hou et al., 2020; Liu et al., 2020; Lv et al., 2020; Tiwari et al., 2020). These 20 miRNAs were miR160, miR164, miR165, miR166, miR167, miR169, miR171, miR172, miR390, miR393, miR396, miR847, miR857, miR156, miR162, miR319, miR395, miR778, miR399, and miR827. We also investigated the expression of their targets. Based on literature search, we only found nine target genes [*Nascent polypeptide-associated complex 1* (*NAC1), NAC2, Homeo-domain leucine zipper (HD_Zip), Indole-3-acetic acid (IAA)-Ala-resistant 3 (IAR3), Nuclear transcription factor Y subunit alpha 2 (NFYA2), Auxin response factor 2 (ARF2), Growth regulator factor 1 (GRF1), Basic helix-loop-helix (bHLH), Squamosa promoter binding protein like 1 (SPL1*)] with nucleotide sequences.

### Statistical Analysis

Depending on different traits, three to six biological replicates were performed. For most physiological and morphology-related traits, six biological replicates were performed. For gene expression analysis, three biological replicates were performed. Statistical software SPSS version 22 was employed to analyze all the data. ANOVA was employed to analyze the significance between the K^+^ deficiency treatment group and the control group. A *p*-value less than 0.05 was marked as * and a *p*-value less than 0.01 was considered a highly significant difference and marked as ∗∗ in graphs.

## Results

### K^+^ Deficiency Significantly Affected Wheat Growth and Biomass

K^+^ deficiency significantly affected wheat seedling aboveground growth and development but had no significant influence on root biomass. At 4 DAT of treatment, the seedlings of the K^+^ deficiency treatment group started to display differences as compared to the control group, including smaller leaves, color change from dark green to light yellow, and smaller plant sizes. After 4 DAT of K^+^ deficiency treatment, the dry plant biomass reduced from 50.78 mg/plant to 39.34 mg/plant with 22.5% decrease as compared to the control group (**Table 1**). After 8 DAT of treatment, the total dry biomass of K^+^ deficiency treatment group was only 53.83 mg/plant, while the control group was 67.22 mg/plant (**Table 1**).

Plant up-ground parts were more sensitive to K^+^ deficiency than the roots (**Table 1**). After 4 DAT and 8 DAT of K^+^ deficiency treatment, the dry shoot weight, dry plant weight, and shoot area were all significantly decreased as compared to the controls. In contrast, there was no significant difference in dry root biomass between the K^+^ deficiency treatment group and the controls (**Table 1**).

**Table 1:** Effect of K^+^ deficiency on plant biomass of wheat seedlings and shoot area at 4/8 days after transfer day (DAT)*.

DAT(d)^&^	Treatments	Dry shoot weight (mg)	Dry root weight (mg)	Dry plant weight (mg)	Shoot area(cm^2^)
4	Control	36.16 + 3.49a	14.62 + 1.61a	50.78 + 4.65a	33.41 + 4.09a
	K^+^ deficiency	24.6 + 2.19b	14.74 + 1.21a	39.34 + 2.91b	28.01 + 3.18b
8	Control	48.1 + 8.13a	19.12 + 4.24a	67.22 + 11.61a	55.03 + 8.18a
	K^+^ deficiency	35.4 + 4.48b	18.43 + 3.03a	53.83 + 7.47b	37.94 + 4.54b

*Different lower-case letters indicate significant difference at P < 0.05 level.

^&^day after transferring wheat seedlings to the treatment medium (DAT).

### K^+^ Deficiency Significantly Affected Wheat Root Development

**Figure 1:**
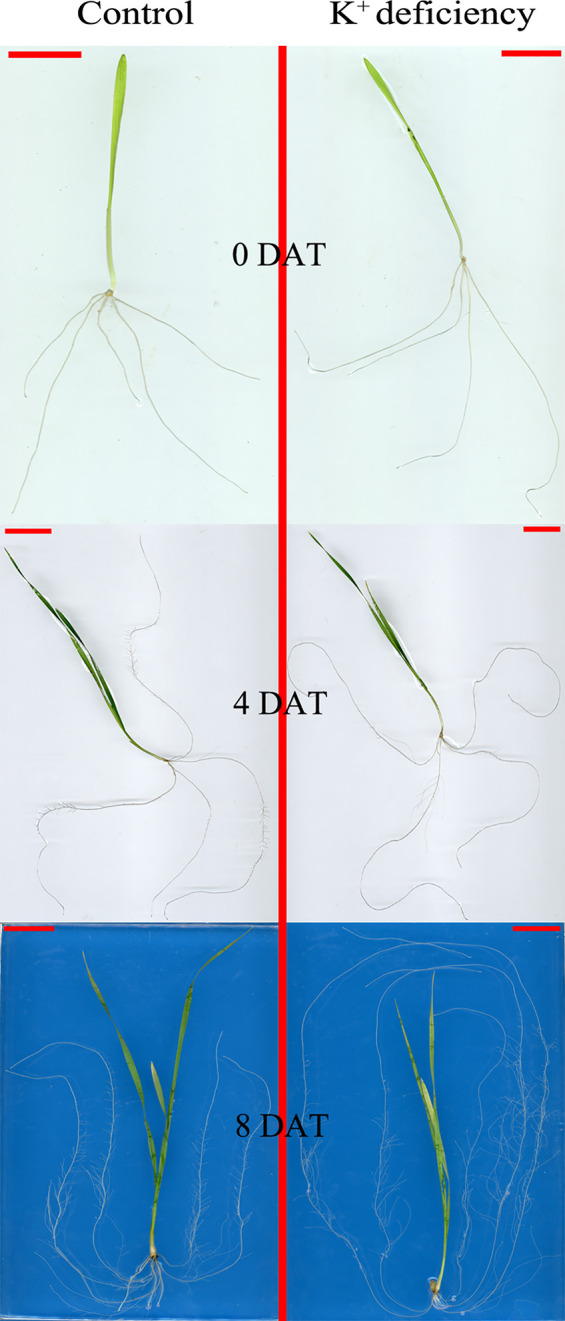
Seedling morphology difference between high K^+^ control group and low K^+^ treatment group at 0, 4 and 8 days after transfer day (DAT).

Although there is no significant effect on root biomass after 8 DAT of K^+^ deficiency treatment, K^+^ deficiency inhibited root development and further affected root growth and development after 8 DAT of treatment. K^+^ deficiency delayed the emergence of seminal roots; after 4 DAT of treatment, there were on average 5.4 seminal roots per plant for the control group, whereas only 4.2 seminal roots per plant in the K^+^ deficiency group. This difference diminished after 8 DAT of treatment where plants in the control group generated 5.5 seminal roots, while plants under K^+^ deficiency treatment produced 5.2 seminal roots. Although the emergence of seminal roots was delayed in the K^+^ deficiency group, the seminal root length, and non-branching zone length were both increased in the K^+^ deficiency group as compared to the controls. Further, the total root length was also increased under the K^+^ deficiency treatment. However, the total root volume and average root diameter were not changed under the K^+^ deficiency treatment (**Table 2** and **Figure 1**).

**Table 2:** Effect of K^+^ deficiency on morphological parameters of wheat seedling roots at 4/8 days after transfer day (DAT).

DAT(d)	Treatments	TRL(cm/plant)	TRS(cm^2^/plant)	TRV(cm^3^/plant)	ARD(mm)	SRN(No/plant)	SR length(cm)		FRN (No.)		BZL(cm)	NBZL(cm)
							cm/SR	cm/plant	No/SR	No/plant		
4	Control	144.60 ± 10.77a	14.53 ± 1.14a	0.12 ± 0.01a	0.32 ± 0.32a	5.4 ± 0.89a	12.34 ± 4.67a	66.63 ± 25.21a	15.89 ± 4.97a	85.8 ± 26.86a	8.13 ± 2.37a	5.29 ± 1.60a
	K^+^ deficiency	153.55 ± 22.56a	15.52 ± 1.57a	0.13 ± 0.02a	0.32 ± 0.32a	4.2 ± 0.45b	13.27 ± 1.91a	55.74 ± 8.04a	10.43 ± 5.86a	43.8 ± 24.64b	6.97 ± 1.58a	8.12 ± 1.15a
8	Control	256.73 ± 46.67 b	22.21 ± 4.12b	0.16 ± 0.03a	0.26 ± 0.26a	5.5 ± 1.29a	13.15 ± 1.19b	72.34 ± 6.55b	34.18 ± 5.45a	188.0 ± 29.97a	10.62 ± 1.68a	4.04 ± 0.22b
	K^+^ deficiency	370.76 ± 63.54a	30.79 ± 4.43a	0.20 ± 0.03a	0.27 ± 0.27a	5.2 ± 0.84a	18.55 ± 2.25a	95.94 ± 11.71a	41.81 ± 7.29a	217.4 ± 37.92a	11.59 ± 0.62a	7.27 ± 0.30a

*Different lower-case letters indicate significant difference at P < 0.05 level. TRL, Total root length: TRS, Total root surface area; TRV, Total root volume; ARD, Average root diameter; SR, Seminal root; FRN, First order roots number; BZL, Branching zone length; NBZL, Non-branching zone length.

### K^+^ Deficiency Affected Wheat Respiration and Root Vitality

Although K^+^ deficiency did not significantly affect root vitality, it affected root and leaf respiration (**Table 3**). The effects were manifested mainly at the early treatment stage. At the 4 DAT of K^+^ deficiency treatment, roots and leaves all displayed higher respiration rate as compared to the controls. As treatment extended to 8 DAT, the respiration rate has no difference between the K^+^ deficiency treatment and the control group, suggesting plant adaption to the K^+^ deficiency condition.

**Table 3:** Effect of K^+^ deficiency on root vitality, root or leaf respiration reflected by oxygen consumption rate at 4/8 days after transfer day (DAT).

DAT(d)	Treatments	Root vitality (OD g^−1^ FW)	Oxygen (O_2_) consuming rate (nmol L^−1^/min/g FW)	
			Root	Leaf
4	Control	2.64 ± 0.31a	204.27 ± 27.65b	712.70 ± 463.90b
	K^+^ deficiency	2.81 ± 0.61a	291.74 ± 45.31a	1371.04 ± 457.35a
8	Control	1.07 ± 0.23a	174.34 ± 24.01a	538.35 ± 153.64a
	K^+^ deficiency	1.33 ± 0.27a	129.23 ± 36.98a	695.13 ± 246.75a

*Different lower-case letters indicate significant difference at P < 0.05 level.

### K^+^ Deficiency Affected Chlorophyll Biosynthesis in Wheat Seedlings

K^+^ deficiency significantly inhibited the biosynthesis of chlorophyll a and b and further affected the whole content of chlorophyll (**Table 4**). Under K^+^ deficiency, the biosynthesis of chlorophyll was reduced by 14.3–20.0% Chlorophyll is one of the major pigments in plant photosynthesis system, responsible for absorbing solar light for plant photosynthesis. Reduction of chlorophyll biosynthesis under K^+^ deficiency results in lower photosynthesis that further affects plant growth and development as well as plant biomass.

**Table 4:** Effect of K^+^ deficiency on chlorophyll at 4/8 days after transfer day (DAT)*.

DAT(d)	Treatments	Chlorophyll a (mg/g)	Chlorophyll b (mg/g)	Chla : Chlb	Chlorophyll content (mg/g)
4	Control	1.27 ± 0.18a	0.35 ± 0.05a	3.63:1a	1.62 ± 0.23a
	K deficiency	1.05 ± 0.07b	0.30 ± 0.02b	3.50:1a	1.35 ± 0.08b
8	Control	1.28 ± 0.11a	0.35 ± 0.03a	3.66:1a	1.63 ± 0.14a
	K deficiency	1.05 ± 0.11b	0.28 ± 0.04b	3.75:1a	1.34 ± 0.15b

*Different lower-case letters indicate significant difference at P < 0.05 level.

### Expression Analysis of miRNAs and Their Target Genes

All 20 tested miRNAs and nine targets were expressed in wheat seedling under the normal culture condition. Among the 20 miRNAs, miR160 was the highest expressed followed by miR164, miR172, miR319, and miR156. Except miR393 and miR167, all other miRNAs were highly expressed compared with the reference gene. All nine miRNA target genes were also expressed with different levels; among them, *HD ZiP* was the highest expressed followed by *GRF1* transcription factor gene. Compared with the reference gene and other tested genes, the expression level of *bHLH* transcription factor gene was relatively low (data not shown).

### K^+^ Deficiency Induced Aberrant Expression of miRNAs and Their Targets

**Figure 2:**
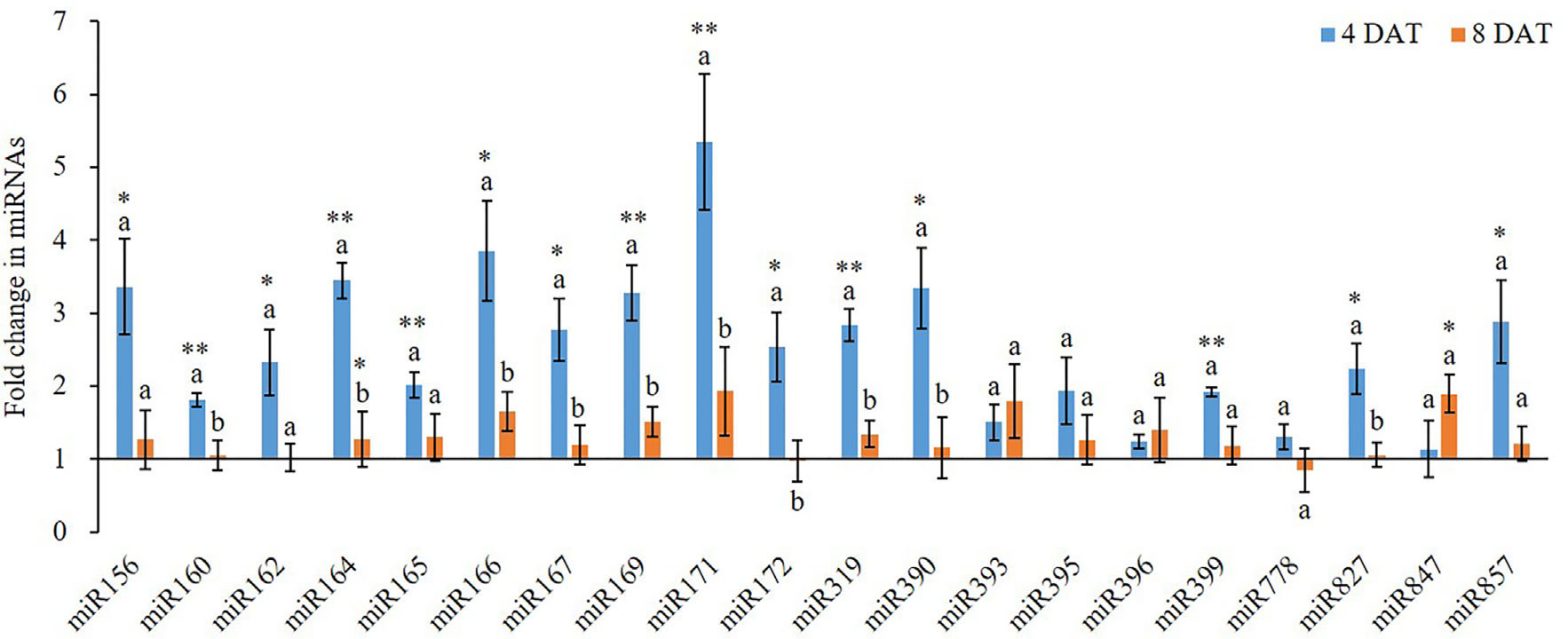
Fold change of miRNA expression between low K^+^ treatment and the controls at 4/8 days after transfer day (DAT). Error bars represent the S.E (n = 3). The * and ** on the bars indicate significant differences of fold change of miRNA expression between low K^+^ treatment and the controls at 4 and 8 DAT at p < 0.05 and p < 0.01, respectively. The different letters on the bars indicate significant differences of fold change of miRNA expression between 4 and 8 DAT according to the LSD test (p = 0.05).

**Figure 3:**
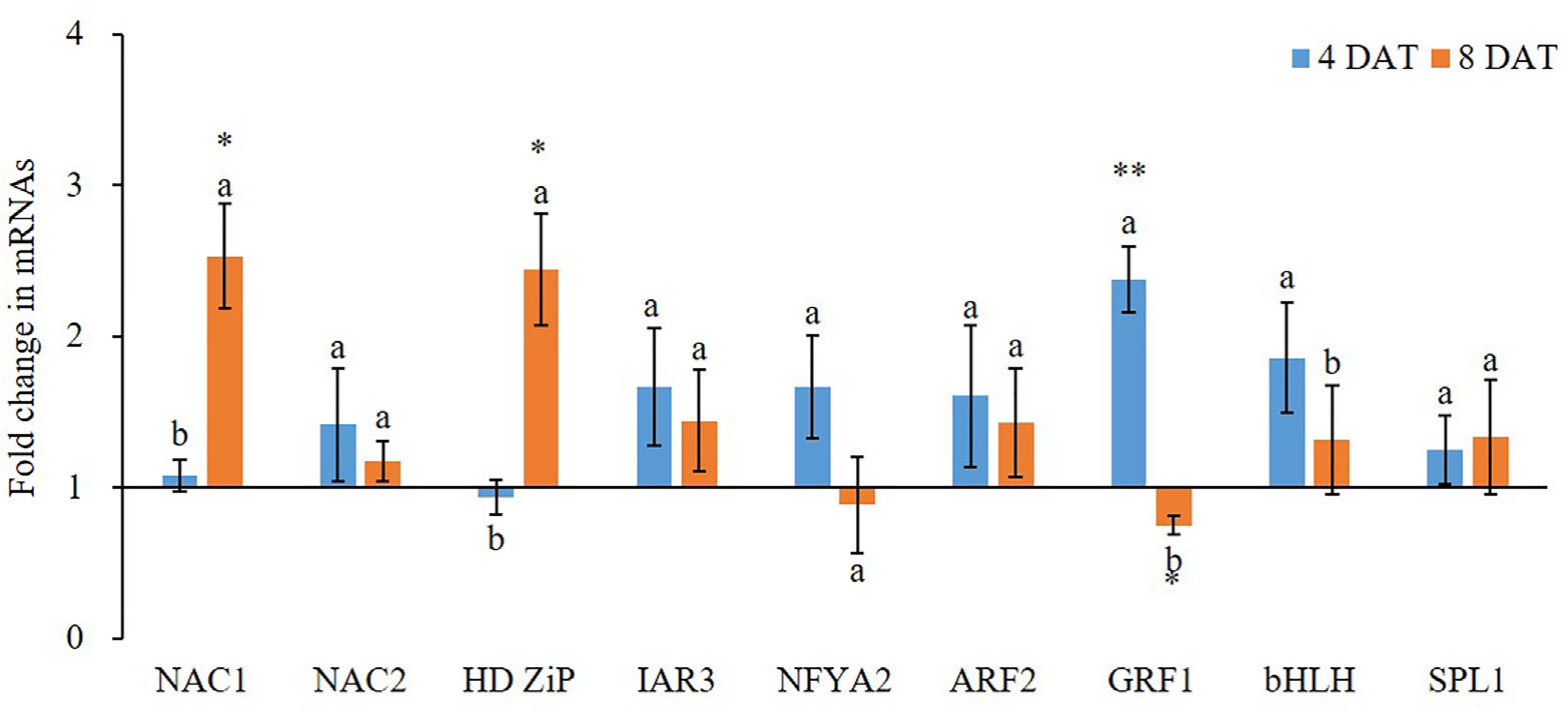
Fold change of miRNA target gene expression between low K^+^ treatment and the controls at 4/8 days after transfer day (DAT). Error bars represent S.E. (n = 3). Highly significant difference was marked as ** to represent a *p* value < 0.01 and as * to represent a *p* value < 0.05 between low K^+^ treatment and the controls. The different letters on the bars indicate significant differences of fold change of miRNA target expression between 4 and 8 DAT according to the LSD test (P = 0.05).

K^+^ deficiency induced aberrant expression of miRNAs and their targets (**Figures 2** and **3**). However, different miRNAs responded to K^+^ deficiency differently (**Figure 2**). At 4 DAT of K^+^ deficiency treatment, miR171 was upregulated the most by 5.35-fold. The expression of miR156, miR164, miR166, miR169, and miR390 was also highly induced with more than 3-fold change. In contrast, when the treatment was extended to 8 DAT, the expression of all tested miRNAs was inhibited compared with 4 DAT, except miR393 (**Figure 2**). Although the expression of the majority of miRNAs was still higher in the K^+^ deficiency treatment group than that in the controls, the fold changes were much smaller than those detected at 4 DAT. At 8 DAT, the expression of miR778 and miR172 was lower in the K^+^ deficiency treatment than that in the controls (**Figure 2**).

K^+^ deficiency treatment also affected the expression of protein-coding genes (**Figure 3**). At 4 DAT, all tested nine potential miRNA targets, except *HD Zip* gene, were highly expressed in K^+^ deficiency treatment compared to that in the controls. Among them, *GRF1* and *bHLH* transcription factor genes were the most highly induced by the K^+^ deficiency treatment as compared to the controls, with 2.38- and 1.86-fold increase, respectively. In contrast, at 8 DAT, the majority of potential miRNA targets were inhibited by K^+^ deficiency treatment except *NAC1* and *HD ZiP* transcription factor genes. Among them, GRF1 transcription factor gene was the most downregulated under the K^+^ deficiency condition; the expression fold change was from 2.38-fold increase at 4 DAT to 0.75-fold decrease at 8 DAT. The expression level of NFYA2 transcription factor gene was also decreased by 47.3% at 8 DAT compared to that at 4 DAT under K^+^ deficiency treatments.

### miRNAs Regulated Wheat Plant Response to K^+^ Deficiency Through Regulation of Protein-Coding Genes

**Figure 4:**
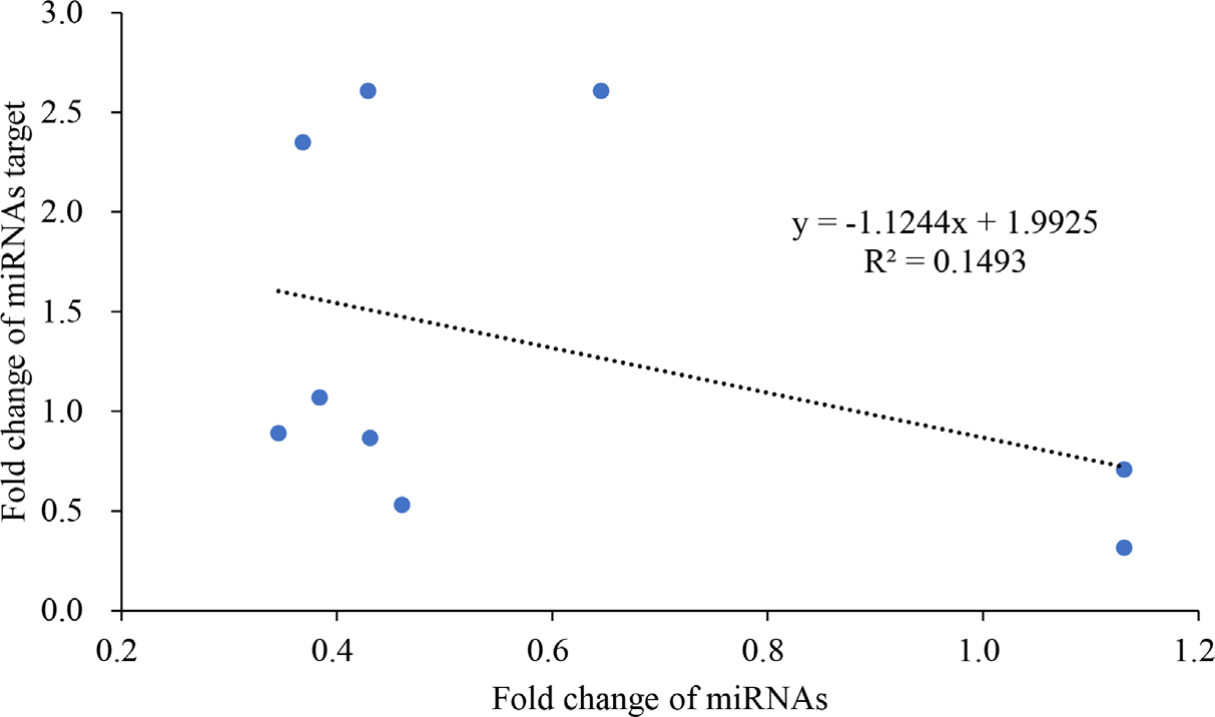
The reverse linear relationship of fold change between miRNAs and their targets.

A miRNA functions through inhibiting its targeted protein-coding gene; therefore, the expression of a miRNA and its gene target should be reversely corelated, *i.e.* if a miRNA is upregulated, the expression of its target should be decreased. However, since there may be secondary responses and other gene regulators besides miRNAs, the expression patterns of miRNAs and their targets are usually complex in real-world scenario where the reverse expression relationship may not exhibit. In this study, we found that, in most cases, K^+^ deficiency treatment inhibited the expression of both miRNAs and their targets. However, the extent of inhibition varies among different miRNAs and their targets. To elucidate the potential relationship between miRNAs and their potential targets, we modeled the relationship between fold changes of miRNAs and their targets from 4 DAT to 8 DAT. The results clearly show that there was a negative linear relationship between miRNAs and their target expressions. The reverse relationship can be described with the equation: y = −1.1244X + 1.9925 (**Figure 4**); the slope is −1.1244. This reverse relationship suggests that the tested miRNAs negatively regulated the expression of their target genes.

## Discussion

Although the effect of fertilizers on plant growth and development has been well studied at different levels, including transcriptomes, few studies were focused on K^+^ in wheat (Krishnasamy et al., 2014; Ruan et al., 2015; Yilmaz et al., 2017; Asif et al., 2018). Yilmaz and colleagues (2017) investigated the impact of K^+^ deficiency on wheat biomass and antioxidative response (Yilmaz et al., 2017). Asif and colleagues (2018) reported the effect of K^+^ deficiency treatment on wheat yield (Asif et al., 2018). In an early study, Ruan and colleagues (2015) compared the gene expression under K^+^ deficiency treatment in two K^+^ deficiency susceptible and tolerant wheat cultivars, in which they also observed that K^+^ deficiency affected root development (Ruan et al., 2015); however many details were not reported. Krishnasamy and co-workers (2014) reported that K^+^ deficiency affected wheat growth, including root growth; after a long term treatment, the root dry weight was greater in adequate K^+^ treatment than that in the K^+^ deficiency in all four tested wheat cultivars (Krishnasamy et al., 2014). However, the majority of these studies were performed in the field without systematic investigation of K^+^ deficiency impacts on root morphology, growth, and development. Our study filled this data gap and showed that K^+^ deficiency delayed seminal root emergence but enhanced seminal root elongation and increased total root length and total root surface area. However, probably due to the short treatment time and quick adaption, we did not observe the significant difference on root biomass between K^+^ deficiency treatment and the controls. It is likely that K^+^ deficiency will eventually inhibit root development and reduce the total fresh and dry root biomass as reported by others (Krishnasamy et al., 2014).

K^+^ is a major nutrient for plants, particularly important for crop yields. However, there is not enough K^+^ in the soil; therefore, for a sustainable crop production, K^+^ fertilizer is always required for agricultural purposes. Deficiency of K^+^ in plants triggers reductions in a myriad of physiological processes, including NO_3_^−^ uptake, photosynthesis, and stomatal conductance; all of those lead to a decrease in crop yield and quality (Pettigrew, 2008). To maximize crop production, it is important to better understand K^+^ deficiency-induced physiological changes and the underlying mechanisms. This study provides a systematic understanding of K^+^ deficiency-induced changes in wheat growth and development, particularly on root development. This study also investigated the miRNA-mediated gene regulation during wheat plant response to different K^+^ concentrations. Overall we found that low K^+^ availability altered the expression of certain miRNAs and their gene targets. Among the 20 tested miRNAs, the expression of 16 miRNAs, except miR393, miR395, miR396 and miR778, was induced by K^+^ deficiency (**Figure 2**) at 4 and/or 8 DAT which was similar to what we found in cotton (Fontana et al., 2020). This may be a molecular mechanism underlying wheat response to K^+^ deficiency to enhance root growth and nutrient uptake. In a review paper, Nguyen et al. (2015) suggested that nitrogen deficiency induced the expression of miR156, miR160, and miR171 in *Arabidopsis* and the expression of miR156, miR160, miR171 miR162, miR167, and miR393 in maize roots; however, nitrogen deficiency inhibited the expression of miR164, miR166, miR169, miR172, miR319, miR396, miR399, miR827, and miR857 in maize roots, the expression of miR169, miR171, miR172, miR319, miR396, miR399, miR827, and miR857 in soybean roots, and the expression of miR169, miR172, miR319, miR396, miR399, miR827, and miR857 in *Arabidopsis* roots (Nguyen et al., 2015). Therefore, the expression patterns of miRNAs were different between K^+^ deficiency and nitrogen deficiency.

*ARF* genes have been shown to play key roles in plant development in many plant species, including *Arabidopsis*. According to previous studies, the *ARF* family genes are targets of several miRNAs, including miR160, miR167 (Wu et al., 2006), and miR390 (Xia et al., 2017). miR160 is an important root development regulator that has been found expressed at high levels in cotton (Pan et al., 2019). miR160 has also been documented to inhibit *ARF-10*, *ARF-16*, and *ARF-17* genes, and to regulate itself by endogenous target mimics (Lin et al., 2015). The miR167 was found to regulate the expression of *ARF6* and *ARF8* in *Arabidopsis* (Wu et al., 2006). The miR390 does not directly target or bind to *ARF* family genes, but has been shown to act as a regulatory factor by targeting *TAS3*, resulting in the production of trans-acting small interfering RNAs (tasiRNA) (Xia et al., 2017). These tasiRNAs then target *ARF* genes, acting to suppress them (Xia et al., 2017). The *HD-ZIP* family of genes have been shown to be targets of both miR165 and miR166. This study shows that K^+^ deficiency affected the expression of ARF transcription factor genes and their corresponding miRNAs.

Genes of the *NAC* transcription factor family have been identified as targets of miR164 in *Arabidopsis*, and that *TaNAC21/22* is a target of miR164 in wheat (Feng et al., 2014). Studies have shown that miR396 targets the Growth regulating factor (*GRF*) gene family in plants and may also act to regulate bHLH74 (Debernardi et al., 2012). miR396 was slightly upregulated at 4 DAT of K^+^ deficiency treatment. At 8 DAT of K^+^ deficiency treatment, *GRF1* was downregulated as opposed to *bHLH* which was slightly upregulated. miR156 targets have been identified in *Arabidopsis* as the *SPL* gene family and have been studied together with miR172 targets in the *AP2-like* family (Wu et al., 2009). The miR156 displayed over 3.3-fold upregulation at 4 DAT of K^+^ deficiency treatment, while miR172 was upregulated by over 2.5-fold. The *SPL1* gene was about 1.3-fold upregulated at 4 and 8 DAT of K^+^ deficiency treatment. miR156 and miR172 were almost equivalently downregulated at 8 DAT of K^+^ deficiency treatment while *SPL1* was upregulated. A study in *Arabidopsis* revealed that miR171 may act antagonistically towards miR156 targeting *SPL2* and *SPL9* genes during the regulation of trichome distribution (Xue et al., 2014; Wang and Guo, 2015). In this study, miR171 showed the highest expression level among all tested miRNAs at the 4 DAT of K^+^ deficiency treatment, and its expression level was reduced at the 8 DAT time point, dropping from 5.35-fold to 1.93-fold upregulation.

*Arabidopsis* root architecture is regulated by *NFYA2* which is targeted by an miR169 isoform (Sorin et al., 2014). In this study, miR169 was nearly 3.3-fold upregulated at 4 DAT of K^+^ deficiency treatment, but its expression was reduced to less than 1.5-fold upregulated at 8 DAT of K^+^ deficiency treatment. The *NFYA2* gene was 2-fold upregulated at 4 DAT of K^+^ deficiency treatment, but by 8 DAT its expression was reduced to a downregulated state. Because NFYA regulated the nitrate transporter expression (Zhao et al., 2011), upregulation of miR169 under K^+^ deficiency further suggests that K^+^ deficiency interferes with the nitrate uptake.

It is well studied that K^+^ deficiency induced aberrant growth and development of plant and altered the expression of protein-coding genes. In this study, we observed a similar phenomenon on wheat growth and development under K^+^ deficiency condition. However, few studies provided detailed findings on root development affected by K^+^ deficiency but no report on miRNA expression during K^+^ deficiency in wheat to date despite that two recent studies reported that K^+^ deficiency altered the expression of certain miRNAs in cotton and barley, respectively (Zeng et al., 2019; Fontana et al., 2020). In this study, we systematically studied the effect of K^+^ deficiency on wheat root development and morphology. Our results show that although K^+^ deficiency did not affect root vitality, it suppressed root development, including root branching, root area, and root size. In addition, K^+^ deficiency delayed seminal root emergence, while it enhanced seminal root elongation, increased total root length and further increased the total root surface area. This suggests that wheat plants attempted to adapt to K^+^ deficiency condition by generating more roots for taking more nutrient from the medium to meet its growth and development needs. Importantly, K^+^ deficiency altered the expression of miRNAs and their targets related to root development. For example, miR171 and miR166 are two miRNAs with the most expression changes after 4 DAT of K^+^ deficiency treatment, which regulate hairy meristem and ZiP transcription factor genes and control lateral root development (Couzigou and Combier, 2016). Under K^+^ deficiency condition, to uptake more K^+^, more miRNAs were synthesized by wheat seedling to enhance root differentiation and development. This results in enhanced growth and development of wheat root to adapt to the low K^+^ environment. However, although the seedlings attempted to adapt to the environment, the long-term K^+^ deficiency will eventually lead to retarded growth and development. In this study, almost all miRNAs and their target genes were upregulated after 4 DAT of K^+^ deficiency treatment, demonstrating a potential adaption mechanism. However, after 8 DAT of K^+^ deficiency treatment, most miRNAs and protein-coding gene expression levels were down which further caused plant growth retardation, indicating the negative impacts of prolonged K^+^ deficiency. Thus, the positive root adaption to K^+^ deficiency at the early stage could be regulated by miRNA-mediated pathway and mechanism which are associated with root development.

## Data Availability Statement

All datasets presented in this study are included in the article.

## Author Contributions

TT, JL, HX, JF, GW, QL, WL, and KD performed experiments. TT, JL, HX, JF, GW, QL, LL, and KD analyzed data. TT, XP, BZ, and ZZ wrote the manuscript. BZ, ZZ, ML, and XP designed the experiment. BZ, ZZ, ML, and XP revised the manuscript. All authors contributed to the article and approved the submitted version.

## Funding

This work was supported by the Key R & D and promotion projects of Henan Province in 2020 (202102110181) and Postgraduate Education Reform and Quality Improvement Project of Henan Province (Award number 2018 No. 23). This project is also supported by the U.S. National Science Foundation (award 1658709 to BZ and XP).

## Conflict of Interest

The authors declare that the research was conducted in the absence of any commercial or financial relationships that could be construed as a potential conflict of interest.

## References

[B1] Asefpour Vakilian K. (2020). Determination of nitrogen deficiency-related microRNAs in plants using fluorescence quenching of graphene oxide nanosheets. Mol. Cell. Probes 52, 101576. 10.1016/j.mcp.2020.101576 32304823

[B2] Asif M. Tunc C. E. Ozturk L. (2018). Changes in yield attributes and K allocation in wheat as affected by K deficiency and elevated CO2. Plant Soil 426, 153–162. 10.1007/s11104-018-3603-z

[B3] Bao H. Chen H. Chen M. Xu H. Huo X. Xu Q. (2019). Transcriptome-wide identification and characterization of microRNAs responsive to phosphate starvation in Populus tomentosa. Funct. Integr. Genomics 19, 953–972. 10.1007/s10142-019-00692-1 31177404

[B4] Couzigou J. M. Combier J. P. (2016). Plant microRNAs: key regulators of root architecture and biotic interactions. New Phytol. 212, 22–35. 10.1111/nph.14058 27292927

[B5] Debernardi J. M. Rodriguez R. E. Mecchia M. A. Palatnik J. F. (2012). Functional specialization of the plant miR396 regulatory network through distinct microRNA-target interactions. PloS Genet. 8, e1002419. 10.1371/journal.pgen.1002419 22242012 PMC3252272

[B6] Feng H. Duan X. Zhang Q. Li X. Wang B. Huang L. (2014). The target gene of tae-miR164, a novel NAC transcription factor from the NAM subfamily, negatively regulates resistance of wheat to stripe rust. Mol. Plant Pathol. 15, 284–296. 10.1111/mpp.12089 24128392 PMC6638668

[B7] Filina V. Grinko A. Ermilova E. (2019). Truncated Hemoglobins 1 and 2 Are Implicated in the Modulation of Phosphorus Deficiency-Induced Nitric Oxide Levels in Chlamydomonas. Cells 8, 947. 10.3390/cells8090947 PMC6770159 31438612

[B8] Fontana J. E. Wang G. Sun R. Xue H. Li Q. Liu J. (2020). Impact of potassium deficiency on cotton growth, development and potential microRNA-mediated mechanism. Plant Physiol. Biochem.: PPB 153, 72–80. 10.1016/j.plaphy.2020.05.006 32480238

[B9] Guo J. Jia Y. Chen H. Zhang L. Yang J. Zhang J. (2019). Growth, photosynthesis, and nutrient uptake in wheat are affected by differences in nitrogen levels and forms and potassium supply. Sci. Rep. 9, 1248. 10.1038/s41598-018-37838-3 30718692 PMC6362105

[B10] Hafsi C. Debez A. Abdelly C. (2014). Potassium deficiency in plants: effects and signaling cascades. Acta Physiol. Plantarum 36, 1055–1070. 10.1007/s11738-014-1491-2

[B11] Hermans C. Hammond J. P. White P. J. Verbruggen N. (2006). How do plants respond to nutrient shortage by biomass allocation? Trends Plant Sci. 11, 610–617. 17092760 10.1016/j.tplants.2006.10.007

[B12] Hou G. Du C. Gao H. Liu S. Sun W. Lu H. (2020). Identification of microRNAs in developing wheat grain that are potentially involved in regulating grain characteristics and the response to nitrogen levels. BMC Plant Biol. 20, 87. 10.1186/s12870-020-2296-7 32103721 PMC7045451

[B13] Jaubert-Possamai S. Noureddine Y. Favery B. (2019). MicroRNAs, New Players in the Plant-Nematode Interaction. Front. Plant Sci. 10, 1180. 10.3389/fpls.2019.01180 31681347 PMC6811602

[B14] Jia Y. B. Yang X. E. Feng Y. Jilani G. (2008). Differential response of root morphology to potassium deficient stress among rice genotypes varying in potassium efficiency. J. Zhejiang Uni. Sci. B 9, 427–434. 10.1631/jzus.B0710636 PMC2367382 18500783

[B15] Jordan-Meille L. Martineau E. Bornot Y. Lavres J. Abreu-Junior C. Domec J. C. (2018). How does water-stressed corn respond to potassium nutrition? A shoot-root scale approach study under controlled conditions. Agriculture 8, 180. 10.3390/agriculture8110180

[B16] Jung J. Y. Shin R. Schachtman D. P. (2009). Ethylene mediates response and tolerance to potassium deprivation in Arabidopsis. Plant Cell 21, 607–621. 10.1105/tpc.108.063099 19190240 PMC2660615

[B17] Kellermeier F. Chardon F. Amtmann A. (2013). Natural variation of Arabidopsis root architecture reveals complementing adaptive strategies to potassium starvation. Plant Physiol. 161, 1421–1432. 10.1104/pp.112.211144 23329148 PMC3585606

[B18] Kellermeier F. Armengaud P. Seditas T. J. Danku J. Salt D. E. Amtmann A. (2014). Analysis of the root system architecture of Arabidopsis provides a quantitative readout of crosstalk between nutritional signals. Plant Cell 26, 1480–1496. 10.1105/tpc.113.122101 24692421 PMC4036566

[B19] Khan G. A. Declerck M. Sorin C. Hartmann C. Crespi M. Lelandais-Brière C. (2011). MicroRNAs as regulators of root development and architecture. Plant Mol. Biol. 77, 47–58. 10.1007/s11103-011-9793-x 21607657

[B20] Kim M. J. Ciani S. Schachtman D. P. (2010). A peroxidase contributes to ROS production during Arabidopsis root response to potassium deficiency. Mol. Plant 3, 420–427. 10.1093/mp/ssp121 20139158

[B21] Krishnasamy K. Bell R. Ma Q. (2014). Wheat responses to sodium vary with potassium use efficiency of cultivars. Front. Plant Sci. 5, 631. 10.3389/fpls.2014.00631 25426133 PMC4227480

[B22] Kulcheski F. R. Côrrea R. Gomes I. A. de Lima J. C. Margis R. (2015). NPK macronutrients and microRNA homeostasis. Front. Plant Sci. 6, 451. 10.3389/fpls.2015.00451 26136763 PMC4468412

[B23] Li C. Zhang B. (2016). MicroRNAs in Control of Plant Development. J. Cell. Physiol. 231, 303–313. 10.1002/jcp.25125 26248304

[B24] Lin Y. Lai Z. Tian Q. Lin L. Lai R. Yang M. (2015). Endogenous target mimics down-regulate miR160 mediation of ARF10, -16, and -17 cleavage during somatic embryogenesis in Dimocarpus longan Lour. Front. Plant Sci. 6, 956. 10.3389/fpls.2015.00956 26594219 PMC4633511

[B25] Liu Z. W. Li H. Liu J. X. Wang Y. Zhuang J. (2020). Integrative transcriptome, proteome, and microRNA analysis reveals the effects of nitrogen sufficiency and deficiency conditions on theanine metabolism in the tea plant (Camellia sinensis). Horticult. Res. 7, 65. 10.1038/s41438-020-0290-8 PMC7192918 32377356

[B26] Lv L. Yu K. Lü H. Zhang X. Liu X. Sun C. (2020). Transcriptome-wide identification of novel circular RNAs in soybean in response to low-phosphorus stress. PloS One 15, e0227243. 10.1371/journal.pone.0227243 31961887 PMC6974154

[B27] Maathuis F. J. M. (2009). Physiological functions of mineral macronutrients. Curr. Opin. Plant Biol. 12, 250–258. 10.1016/j.pbi.2009.04.003 19473870

[B28] Nguyen G. N. Rothstein S. J. Spangenberg G. Kant S. (2015). Role of microRNAs involved in plant response to nitrogen and phosphorus limiting conditions. Front. Plant Sci. 6, 629. 10.3389/fpls.2015.00629 26322069 PMC4534779

[B29] Pan X. Nichols R. L. Li C. Zhang B. (2019). MicroRNA-target gene responses to root knot nematode (Meloidogyne incognita) infection in cotton (Gossypium hirsutum L.). Genomics 111, 383–390. 10.1016/j.ygeno.2018.02.013 29481843

[B30] Paux E. Sourdille P. Salse J. Saintenac C. Choulet F. Leroy P. (2008). A physical map of the 1-gigabase bread wheat chromosome 3B. 322, 101–104. 10.1126/science.1161847 18832645

[B31] Pettigrew W. T. (2008). Potassium influences on yield and quality production for maize, wheat, soybean and cotton. Physiol. Plantarum 133, 670–681. 10.1111/j.1399-3054.2008.01073.x 18331406

[B32] Rengel Z. Damon P. M. (2008). Crops and genotypes differ in efficiency of potassium uptake and use. Physiol. Plantarum 133, 624–636. 10.1111/j.1399-3054.2008.01079.x 18397208

[B33] Ruan L. Zhang J. Xin X. Zhang C. Ma D. Chen L. (2015). Comparative analysis of potassium deficiency-responsive transcriptomes in low potassium susceptible and tolerant wheat (*Triticum aestivum* L.). Sci. Rep. 5, 10090. 10.1038/srep10090 25985414 PMC4650753

[B34] Shin R. Schachtman D. P. (2004). Hydrogen peroxide mediates plant root cell response to nutrient deprivation. Proc. Natl. Acad. Sci. U.S.A. 101, 8827–8832. 10.1073/pnas.0401707101 15173595 PMC423280

[B35] Sorin C. Declerck M. Christ A. Blein T. Ma L. Lelandais-Brière C. (2014). A miR169 isoform regulates specific NF-YA targets and root architecture in Arabidopsis. New Phytol. 202, 1197–1211. 10.1111/nph.12735 24533947

[B36] Tiwari J. K. Buckseth T. Zinta R. Saraswati A. Singh R. K. Rawat S. (2020). Genome-wide identification and characterization of microRNAs by small RNA sequencing for low nitrogen stress in potato. PloS One 15, e0233076. 10.1371/journal.pone.0233076 32428011 PMC7237020

[B37] Wang J. J. Guo H. S. (2015). Cleavage of INDOLE-3-ACETIC ACID INDUCIBLE28 mRNA by microRNA847 upregulates auxin signaling to modulate cell proliferation and lateral organ growth in Arabidopsis. Plant Cell 27, 574–590. 10.1105/tpc.15.00101 25794935 PMC4558675

[B38] Wu M. F. Tian Q. Reed J. W. (2006). Arabidopsis microRNA167 controls patterns of ARF6 and ARF8 expression, and regulates both female and male reproduction. Development 133, 4211–4218. 10.1242/dev.02602 17021043

[B39] Wu G. Park M. Y. Conway S. R. Wang J. W. Weigel D. Poethig R. S. (2009). The sequential action of miR156 and miR172 regulates developmental timing in Arabidopsis. Cell 138, 750–759. 10.1016/j.cell.2009.06.031 19703400 PMC2732587

[B40] Xia R. Xu J. Meyers B. C. (2017). The Emergence, Evolution, and Diversiﬁcation of the miR390-TAS3-ARF Pathway in Land Plants. Plant Cell 29, 1232–1247. 10.1105/tpc.17.00185 28442597 PMC5502456

[B41] Xue X. Y. Zhao B. Chao L. M. Chen D. Y. Cui W. R. Mao Y. B. (2014). Interaction between two timing microRNAs controls trichome distribution in Arabidopsis. PloS Genet. 10, e1004266. 10.1371/journal.pgen.1004266 24699192 PMC3974651

[B42] Yang Z. Wang Z. Yang C. Yang Z. Li H. Wu Y. (2019). Physiological responses and small RNAs changes in maize under nitrogen deficiency and resupply. Genes Genomics 41, 1183–1194. 10.1007/s13258-019-00848-0 31313105

[B43] Yilmaz O. Kahraman K. Ozgur R. Uzilday B. Turkan I. Ozturk L. (2017). Growth performance and antioxidative response in bread and durum wheat plants grown with varied potassium treatments under ambient and elevated carbon dioxide. Environ. Exp. Bot. 137, 26–35. 10.1016/j.envexpbot.2017.01.012

[B44] Zeng J. Ye Z. He X. Zhang G. (2019). Identification of microRNAs and their targets responding to low-potassium stress in two barley genotypes differing in low-K tolerance. J. Plant Physiol. 234-235, 44–53. 10.1016/j.jplph.2019.01.011 30665047

[B45] Zhang B. Wang Q. (2015). MicroRNA-based biotechnology for plant improvement. J. Cell. Physiol. 230, 1–15. 10.1002/jcp.24685 24909308

[B46] Zhang B. Pan X. Cannon C. H. Cobb G. P. Anderson T. A. (2006). Conservation and divergence of plant microRNA genes. Plant J.: Cell Mol. Biol. 46, 243–259. 10.1111/j.1365-313X.2006.02697.x 16623887

[B47] Zhang Z. Wang Q. Li Z. Duan L. Tian X. (2009). Effects of Potassium Deficiency on Root Growth of Cotton Seedlings and Its Physiological Mechanisms. Acta Agron. Sin. 35, 718–723. 10.1016/S1875-2780(08)60079-6

[B48] Zhang B. (2015). MicroRNA: a new target for improving plant tolerance to abiotic stress. J. Exp. Bot. 66, 1749–1761. 10.1093/jxb/erv013 25697792 PMC4669559

[B49] Zhang B. H. Unver T. (2018). A critical and speculative review on microRNA technology in crop improvement: Current challenges and future directions. Plant Sci. 274, 193–200. 10.1016/j.plantsci.2018.05.031 30080603

[B50] Zhao M. Ding H. Zhu J.-K. Zhang F. Li W.-X. (2011). Involvement of miR169 in the nitrogen-starvation responses in Arabidopsis. New Phytol. 190, 906–915. 10.1111/j.1469-8137.2011.03647.x 21348874 PMC3586203

